# The State Society and Association Not Rivals!

**Published:** 1903-09

**Authors:** 


### The State Society and Association Not Rivals !

IN the last issue of the JOURNAL, we made the statement that, although there was rivalry between the several state medical societies of the State of New York, there was no rivalry or competition between the state society of 1806 and the state association of 1900, because the members of the association did not constitute a new sect in medicine. This statement will bear examination. Some of our readers may recall conversations or addresses, which seem to indicate the contrary to the above proposition, thus leading to the belief that there is the most active rivalry between these two bodies; in fact, that the very existence of the association, and the organisation of its county branches was, and is, a distinct rival movement, and that the chief end and purpose of the association is to rival, to embarrass, to coerce, to drive out the state society and the county societies, as incompetent, absolutely effete organisations.

The JOURNAL does not desire to maintain that individuals have not now and then been of the above mind, or something like it; or may have had such thoughts and given similar expressions to them. We are not concerned with the vagaries of individuals, but are dealing with a medical movement which finds its expression in statutory law, with corporations which may be considered and tested by their articles of incorporation, holding that the minds of the incorporators can be studied best in those formal declarations of intentions and purposes, now crystalised and preserved in statute and charter.

After considerable study of the histories and charters of the state society and the state association, we asseverate without fear of successful challenge, that rivalry or competition between these two bodies is impossible, for the following reasons:

1. The theory of the state regarding doctors has been, and is that in the commonwealth there is one medical profession and that was organised into state and county societies by the law of 1806, and its amendments. That subsequently,—namely, in 1857 and in 1865,—it became necessary to organise sects in medicine and it was done, but without violating the original principle that in the state there was one medical profession. That since 1865, there has been no demand for recognition of another and later sect, and no such sect has been recognised or created, and no such sect now exists. Even the sects created in 1857 and 1865 are now practically abolished as such, by the union created under practice act of 1890 and its amendments.

2. The law organising the medical profession and establishing the state society, is the chief corner stone of the public health law of the state, while the state association is unknown and unmentioned in that law.

3. The state society is an inclusive representative medical body, incorporated "for the purpose of regulating the practice of physic and surgery in this state", by act of 1806, which declared itself to be "a public act" while the state association is a private, exclusive corporation; membership in the county societies of 1806 is obligatory, whereas membership in the county associations of 1900 is optional.

4. Chapter 452, laws of 1900, incorporating the state association neither repeals, amends, nor mentions any part of the public health law, or the law authorising state and county medical societies. It creates a private corporation having scientific, social and business purposes, but without a vestige of the general executive and administrative powers enjoyed by the state society.

For these and other reasons that are not essential to consider now, the state society and the state association cannot be regarded as corporations of the same class; the former is public, the latter private; they have different methods, aims and purposes, different planes and orbits; moreover, by direction of their respective charters they are bodies distinct and apart and can never be rivals nor competitors.

